# Transparent wood composite prepared from two commercially important tropical timber species

**DOI:** 10.1038/s41598-023-42242-7

**Published:** 2023-09-09

**Authors:** M. C. Anish, Krishna K. Pandey, Rakesh Kumar

**Affiliations:** 1 Institute of Wood Science and Technology, Bengaluru, Karnataka India; 2 Department of Forest Products and Utilization, College of Forestry, Kerala Agricultural University, Thrissur, Kerala India

**Keywords:** Environmental sciences, Materials science

## Abstract

Transparent wood (TW) has garnered significant global attention due to its unique properties. In this study, TW composites were fabricated using two timber species of different density classes: *Ailanthus triphysa* (common name: Ailanthus wood) and *Hevea brasiliensis* (common name: Rubberwood). Sodium hydroxide (NaOH) and Hydrogen peroxide-based alkali method was used to modify the lignin in these veneer samples, producing a white cellulose template with a fully intact hierarchical cell structure. Subsequently, a cost-effective thermosetting unsaturated polyester resin (UPR) was infiltrated into the redesigned framework and polymerized to create rigid nanostructured transparent composites. High optical haze (of 94% and 89%) and favourable light transmittance of 59 and 55 percent were exhibited by the UPR-TW composites made from rubberwood and ailanthus wood, respectively. TW was characterised using Scanning electron microscopy and Fourier-transform infrared spectroscopy. The mechanical properties of TW were measured and compared with those of natural wood and pure-polymer. Furthermore, the anisotropic light diffusion behaviour displayed by TW in accordance with the fibre orientation indicates the utility of material as a potential light shaping device. Therefore, a cost-effective and commercially viable strategy to fabricate multipurpose TW composites using a combination of lesser-known timber species (LKTS) and UPR resin was successfully demonstrated.

## Introduction

Wood is a renowned biological resource closely connected to human civilisation since time immemorial. Though solid wood is highly preferred for several applications, the insatiable global demand for the same has compelled mankind to go for alternative sustainable solutions^1,2^. Therefore, the issue related to raw material shortage and the limitations associated with the traditional utilization of natural wood has been tackled through emergence of wood composites^3^. Wood composites offer a variety of uses and advantages by combining the best qualities of both natural and synthetic materials^4–6^. Transparent wood (TW) is one of the novel concepts among such wood polymer composites which possess unique optical properties making it a fit input material for variety of applications^7,8^. TW combines the physical properties of wood with distinctive light transmittance characteristic^9^.

In TW preparation, the light-absorbing component of wood, called lignin was removed to a significant extent to infuse it with a transparent polymer having refractive index (RI) matching to that of cellulose substrate^10^. During the entire process of preparation, the structural integrity of the wood was preserved to allow distinct levels of light transmittance^11,12^. TW possesses several features such as mechanical strength, lightweight, shatterproof, thermal insulation, along with high light transmittance and haze^13^. These attributes ascribed to TW was found to be further enhanced through the incorporation of different species, sample thickness, multiple substrate orientations, polymer varieties, nanomaterials etc.^14–20^. The potential uses of TW in architecture and construction are immense. It can be used as windows, light diffusors, and roof top glazing materials in energy efficient buildings^21,22^. Additionally, it could be utilized to offer a typical visual appeal to furniture and designs such tables, lampshades, and ornamental panels. TW can also find use in solar energy and light-guiding technology^15^.

Despite being a promising material, there are major challenges to be addressed before it is widely used. These difficulties include enhancing the manufacturing process's scalability, fine-tuning of the material’s transparency and durability, and lowering of production costs. The present study addresses these issues with defined objectives such as (a) to expand the scope of TW fabrication through the utilization of lesser known, commercially important timber species (LKTS) thereby reducing the pressure on traditional timber species, (b) incorporation of low-cost polymer instead of conventionally used expensive polymer types to reduce the overall cost of TW production.

## Experimental section

### Materials

The basic substrates i.e., tangential veneers of two species one under the low-density category (i.e., Ailanthus wood density—330–435 kg m^−3^) and the other a medium density timber (i.e., Rubberwood density—density 560–640 kg m^−3^) were collected from the plywood and matchwood industries of Kerala, India. The chemicals such as sodium hydroxide (NaOH) (SD fine-chemicals limited), disodium salt of ethylenediaminetetraacetic acid (EDTA) (HiMedia), magnesium sulfate (MgSO_4_) (Fischer scientific), and hydrogen peroxide (H_2_O_2_) (SD fine-chemicals limited) were used in the lignin modification of wood. A type of commercially available unsaturated polyester resin (UPR) named as ‘*Roof Light*’ (chiefly consisting of a combination of phthalic anhydride, maleic anhydride, propylene glycol, and methyl methacrylate crosslinked or diluted with styrene monomers in the ratio 60:40), methyl ethyl ketone peroxide (MEKP) initiator and cobalt octoate (Co. Oct.) accelerator were supplied by Annapurna agencies, Bangalore. Based on the repeated trial and error experiments, a formulation consisting of 0.5% Co. Oct. and 0.25% MEKP in 100 gm of resin was found to be ideal for TW preparation and processing.

### Fabrication of transparent wood composite

**Figure 1:**
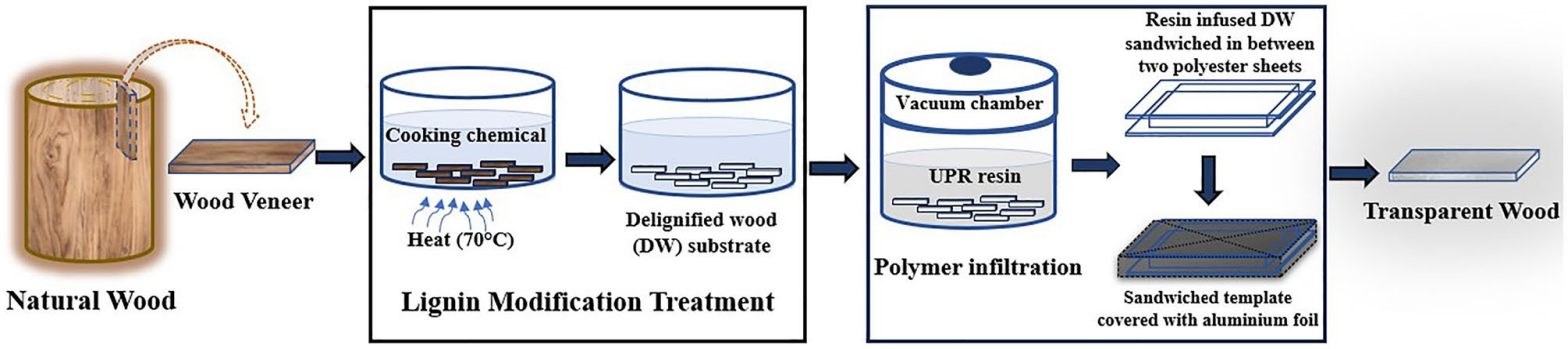
Schematic depiction of processing steps to fabricate transparent wood composites (TW) from natural wood.

Figure 1 shows a schematic representation of the TW fabrication process. Wood veneers of dimensions 20 × 20 × 2 mm^3^ (length x width x thickness) were immersed in an aqueous solution of NaOH (3.0 wt%) and EDTA (0.1wt%) and heated at 70 °C for 10 min. Subsequently, MgSO_4_ (0.1 wt%) was added, followed by H_2_O_2_ (4.0 wt%), and continued heating until the substrate become white. This lignin-modified wood (LMW) substrates were boiled in deionized water (DI) to remove the residual chemicals. Then the chemical free samples were repeatedly washed using ethanol followed by acetone as part of dehydration process and subsequently stored in ethanol. The standard method of lignin estimation (Technical Association of Pulp and Paper Industry Standard Method T 222-om-83) was followed to determine the lignin content, before and after lignin modification.

The dehydrated LMW specimens were then impregnated with a UPR resin formulation consisting of 0.5% Co. Oct. and 0.25% MEKP in 100 gm of resin under vacuum and specimens were then sandwiched between two thin transparent polyester sheets (100 microns). Then the sandwiched specimen was packaged in aluminium foil to facilitate uniform polymerization. The polymerization process was completed by heating the infiltrated wood sample in an oven at 50 °C for 6–8 h. The dried transparent wood composites were then carefully peeled off for material characterization.

### Characterization of transparent wood composite

The cross-sectional structures of natural wood veneer (NW), LMW, and TW composites were characterized by using a Scanning Electron Microscope (Gemini Ultra 55 S). The chemical changes on the wood surface during the sequential process of TW fabrication were evaluated by an FTIR spectrometer (PerkinElmer Spectrum Two) equipped with an attenuated total reflectance (ATR) probe. The spectral resolution was 4 cm^−1^ at the rate of 64 scans/measurement. The weight of samples before and after polymer impregnation were measured using an electronic weighing balance (Sartorius weighing balance, model no: BSA2235), and the percentage weight gain (WPG) was estimated using Eq. 1.

1 $$WPG = \frac{{W_{1} - W_{0} }}{{W_{0} }} \times 100$$

where *W*_0_ and *W*_1_ represents mass before and after polymer impregnation, respectively.

An UV–vis spectrophotometer (Shimadzu, UV-MPC-3100) equipped with an integrating sphere was used for the optical transmittance measurement of the composite. Haze measurement was performed according to the “Standard method for haze and luminous transmittance of transparent plastics” (STM D1003). The TPW sample was placed in front of the port of the integrating sphere through which incident light was passed and the spectrum of the light coupled out of the integrating sphere was analysed at the port perpendicular to the incident beam direction. The mechanical properties were evaluated using a universal testing machine (MultiTest 10-i Mechmesin)**.** Standard test method to determine the flexural properties of reinforced plastic composites (ASTM D790) was used for the strength property evaluation of TW.

Evaluation using six number of TW samples with four test repetitions were carried out respectively for transmittance and haze measurement, FTIR analysis, and mechanical property evaluation.

## Results and discussion

### Lignin modification and response

The lignin content of both timber species before and after lignin removal process was determined. The untreated rubberwood contained 22 ± 0.5% lignin while ailanthus wood veneer was estimated to have 28 + 1.02% acid insoluble lignin prior to lignin modification. These values are consistent with previous findings, which reported a range of 20–35 wt% lignin in wood matrix^23,24^. The time required for delignification to produce a cellulose-rich white template varied according to species and treatment conditions. In certain cases, wood samples need to be cooked/treated until they appear to be white^25,26^. In the present study, substrate with satisfactory brightness was obtained after 4 h of lignin modification from rubberwood. At this stage bleached rubberwood was found to have 57% of retained lignin. Whereas for ailanthus wood, 69% lignin retaining white cellulose-rich template was obtained after 3 h lignin modification reaction. Therefore, here we have optimized the treatment duration suitable for TW preparation from two wood species representing different density classes and lignin content. In addition, we calculated the percentage reduction in lignin content in each species consequent to lignin removal. Partial lignin removal strategy reported by Li et al. was followed in the present work to obtain the delignified samples with well-preserved microstructures^25^. The greater amount of retained lignin inside the bleached provided ample strength to the wood template to facilitate its further processing. Rao et al. followed a similar protocol for lignin removal and reported optimum delignification in poplar wood samples with 88% retention of lignin^27^. Identical strategies involving the selective removal of colour inducing chromophores without drastically removing constituent lignin inside the wood was replicated elsewhere to acquire suitable substrate for TW preparation^20,28–31^. Our study thus validated the efficiency of sodium hydroxide-based lignin modification method through its application in the preparation of substrate from two commercially important timber species. As the chosen species represents two distinct density classes of timbers (i.e. medium and low), the present work further broadens the range of species to which the treatment can be applied.

### Scanning electron microscopy

**Figure 2:**
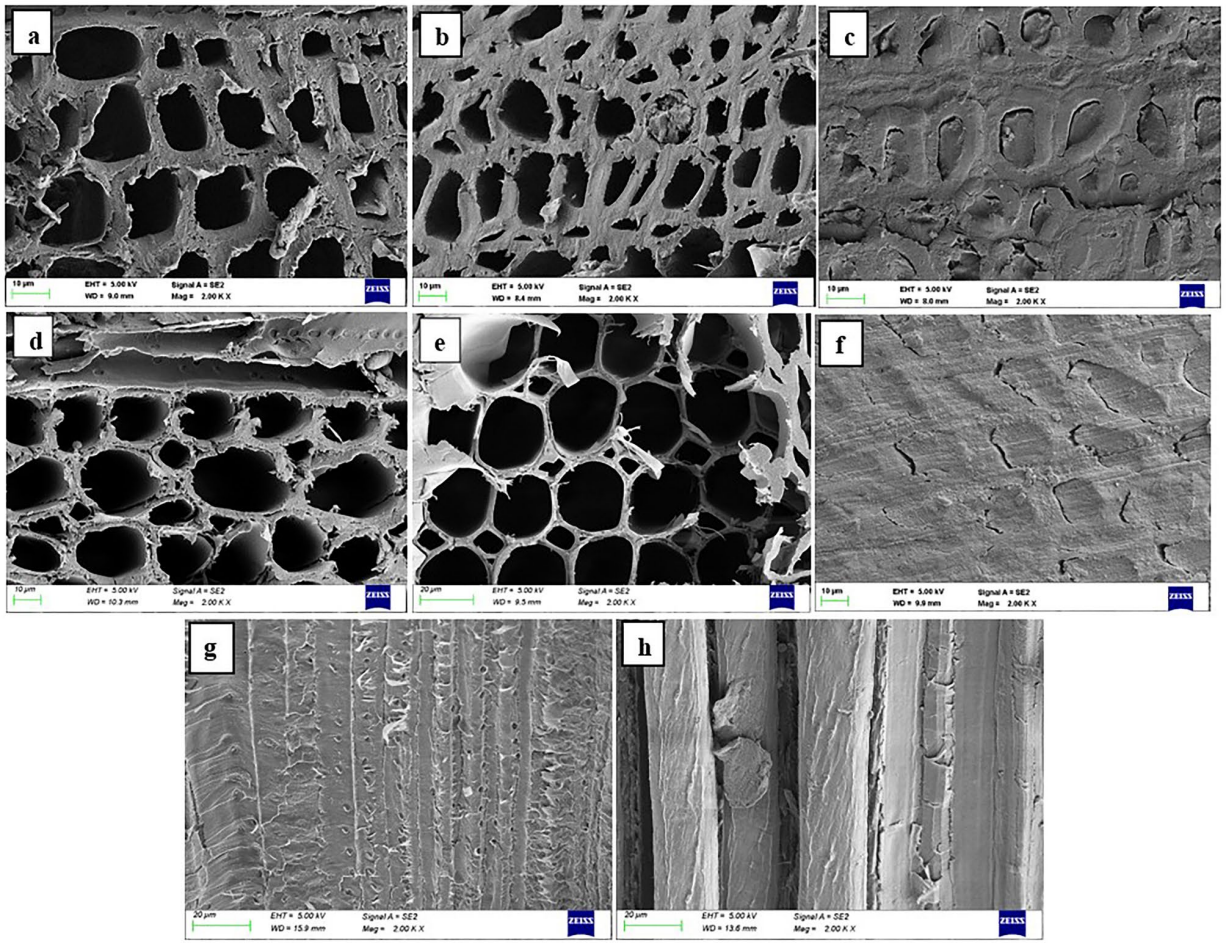
SEM micrographs of transverse sections (TS) of: (**a**) Rubberwood veneer, (**b**) lignin modified rubberwood, (**c**) rubberwood TW, (**d**) ailanthus wood veneer, (**e**) lignin modified ailanthus wood, (**f**) ailanthus wood TW; longitudinal sections of (**g**) rubberwood TW, and (**h**) ailanthus wood TW.

The SEM image of specimens of NW, LMW, and TW composite made using the above-mentioned species are illustrated in Fig. 2. The cell wall structure of natural wood prior to lignin modification was found visibly intact in both the wood species. These pore structures enable transportation of water and mineral inside tree trunk^32^. After partial lignin removal the cell structures had undergone slight thinning and deformation yet retaining its form. The removal of lignin and hemicellulose caused samples to show evidence of cellular level shrinkage as a result of cell wall delamination, albeit to a limited amount^25,27,33^. These largely preserved cellular channels after partial lignin removal facilitates fast infiltration of polymers under vacuum^14^. The filled cell spaces indicate complete infiltration of polymer throughout the bulk of substrate. The SEM images of cross as well as longitudinal sections of TW clearly depicts the uniform distribution of UPR inside the cell lumen of both species after polymerization. The minute cracks evident in the TW cross-sectional image indicate the possible existence of interfacial gaps between the polymer in the lumen and the wood cell. The formation of interfacial debonding fractures is primarily ascribed to poor compatibility between the wood cell wall and polymer and/or volume shrinkage of UPR resin during polymerization^7,34^. Further improvements through various treatments such as acetylation and succinylation were proposed as additional treatment in TW fabrication in order to enhance cell wall—polymer interfacial bonding^35,36^.

### Optical property of TW

**Figure 3:**
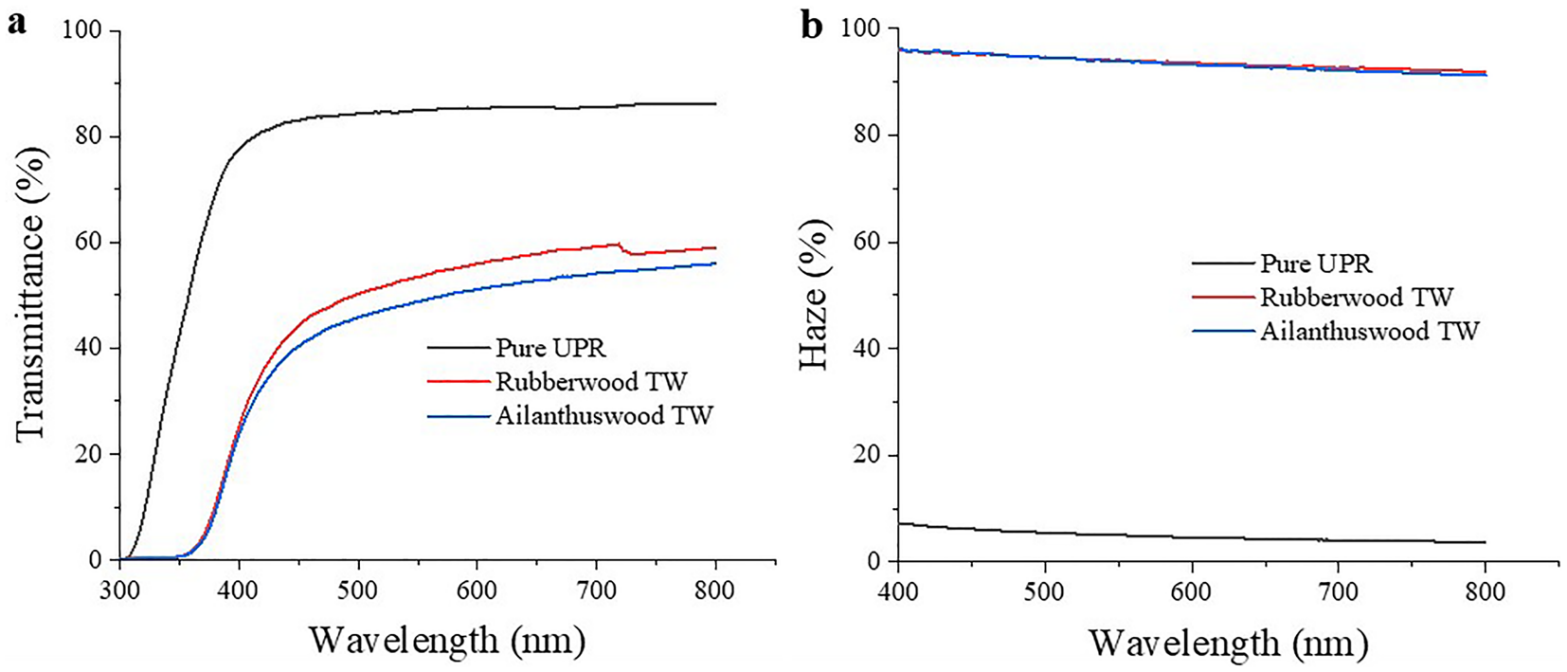
(**a**) Optical transmittance and (**b**) haze of 2 mm thick sheets of pure UPR, Rubberwood TW and Ailanthus wood TW.

The optical transmittance and haze of TW composite prepared using ailanthus and rubberwood are illustrated in Fig. 3. The transmittance exhibited by rubberwood TW was 59% and that of low-density Ailanthus TW was 55% (at λ = 720 nm) and at a wavelength of 550 nm the respective transmittance values becomes 54% and 50%. Relative decrease in transmittance values observed below 450 nm was attributed to the residual lignin in the cellulose substrate^17^. The average optical haze value exhibited by both TW composites were 94% and 89%, respectively. Wu et al. studied optical property of TW fabricated using six different species and found that transmittance and haze of polymer impregnated wood varies with the nature of the species^18^. Similarly in another study, rubberwood of 0.5 mm thickness impregnated with polymers such as polyvinyl-pyrrolidone (PVP), polyvinyl alcohol (PVOH), and methyl methacrylate (MMA) resulted in TW having optical transmittance of 73.4%, 76.6% and 64.6%^37^. Whereas in our case, we have used rubberwood veneers of greater thickness (2 mm) and a different polymer (UPR) to prepare TW to achieve a maximum transmittance of 59%. It was observed that in TW composites, greater thickness leads to proportionate increase of solid–solid interfaces inside the composite matrix. This in turn results in increased attenuation of light to reduce overall transmittance^15^. The phenomenon of reduction in transmittance in relation to the increasing composite thickness was explained in accordance with the Beer–Lambert law^7,34,38^.

Zhu et al. reported 80% transmittance for 2 mm thick basswood based transparent wood^14^. The present work also indicates that apart from the thickness parameter the wood texture and polymer type also have profound influence on the optical characteristic of TW. The combination of the veneer substrates obtained from coarse textured wood when impregnated with UPR resin resulted in TW with lower transmittance and high haze. Though complete infiltration of polymers into the wood cell lumens was evident from Fig. 2c and f, the adverse influence of minute interface gaps due to polymer volume shrinkage could not be ruled out. The increased polymer volume shrinkage observed during UPR polymerization has been connected to the formation of interfacial gaps^39^. Microcracks in polymer composites are also generated by thermal expansion coefficient incompatibility between the reinforcement and the matrix, and cure shrinkage in thermosetting matrices^40,41^. The presence of air in the cell wall-polymer interfacial gaps elevated the refractive index mismatch within the composite causing the hampering of straight light passage through the matrix. Such interfacial deboning inside TW composites creates optical heterogeneity resulting in increased light scattering to ultimately reduce its optical transmittance^34,40,42^.

The percentage weight gain (WPG) of each sample impregnated with UPR resin reflects the degree of polymer infiltration in the respective samples. In response to delignification reaction, a significant weight loss of ≈ 60% for rubberwood and ≈ 70% for ailanthus wood was found compared to the pristine wood samples. Following the polymer impregnation, the WPG value for rubberwood TW is about 200% and ≈ 250% for ailanthus wood TW which justifies the SEM results indicating the adequate infiltration of polymer throughout the bulk of delignified wood substrates. The results are in conformity with the earlier published works where they have reported around 200% weight gain for 1.5 mm poplar TW^43^. The degree of polymer impregnation is an important stage in TW fabrication because it minimises the refractive index contrast inside the composite matrix, resulting in higher optical transmittance^7,11^.

### FTIR analysis

**Figure 4:**
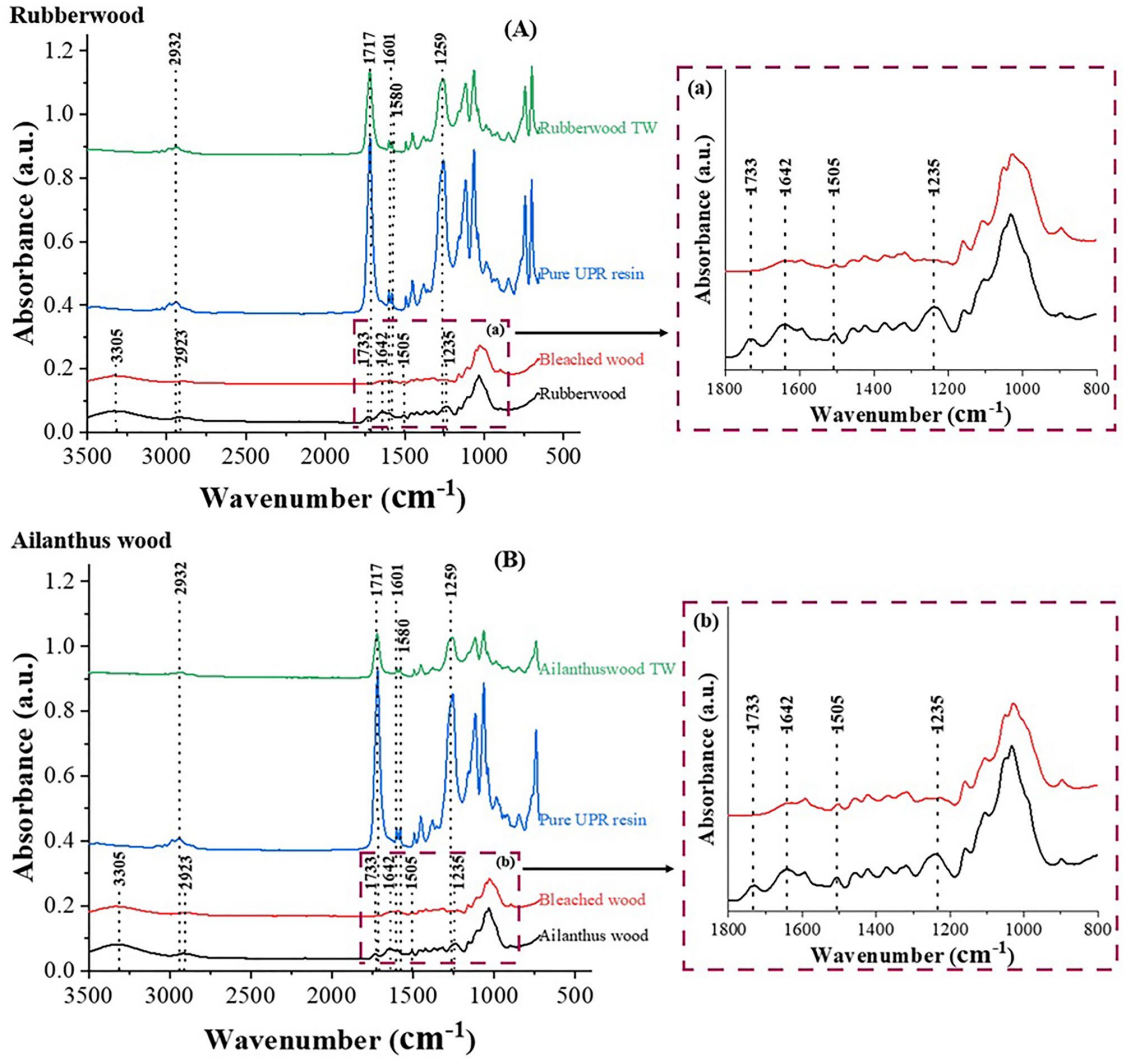
FTIR spectra of (**A**) rubberwood, bleached rubberwood, unsaturated polyester resin (UPR), and rubberwood TW; (**a**) Magnified view of the spectral region showing preservation of lignin and other chemical changes in rubberwood after lignin modification and (**B**) ailanthus wood, bleached ailanthus wood, unsaturated polyester resin (UPR), and ailanthus TW; (**b**) Magnified view of the spectral region showing preservation of lignin and other chemical changes in ailanthus wood after lignin modification.

Figure 4 presents the FTIR spectra of natural wood, lignin modified wood (bleached wood), UPR, and TW composites made from two timber species. The characteristic absorption peaks of normal wood include –OH band at 3305 cm^−1^, the C–H stretching at 2923 cm^−1^, C=O stretching at 1733 cm^−1^ (acetyl groups in hemicelluloses), 1642 cm^−1^ (C=O stretching vibrations), 1505 cm^−1^ (aromatic skeletal vibrations in lignin), 1235 cm^−1^ (syringyl ring and CO stretch in lignin and hemicellulose) etc.^44–47^. It could be noted that after lignin modification the internal lignin groups were almost removed from both rubberwood and ailanthus wood samples. The reduction in intensities of bands at 1505 cm^−1^ and 1235 cm^−1^ corresponding to lignin and hemicellulose peak at 1733 cm^−1^ justify the above observation. The results are in conformity with the earlier published works which attributed the disappearance of respective peaks to lignin degradation in wood as a result of delignification treatment^48,49^.

The FTIR analysis of UPR and TW from both species showed almost identical spectra with respect to the characteristic bands. The peak at 2932 cm^−1^ corresponds to stretching vibrations of CH groups. The same absorption band was still existing with reduced intensity in TW spectra. The carbonyl stretching at 1717 cm^−1^, ring stretching at 1601 cm^−1^ and 1580 cm^−1^, asymmetric stretching vibration at 1259 cm^−1^ representing aliphatic–aromatic ether C–O–C etc. are present with both UPR and TW spectra. The identical spectra of UPR and TW is chiefly due to the encapsulation of the delignified wood substrates by UPR. Similar results were reported for snail shell/polyester composites where minimum influence of fillers was observed on polyester functional groups^50^.

### Mechanical properties

**Figure 5:**
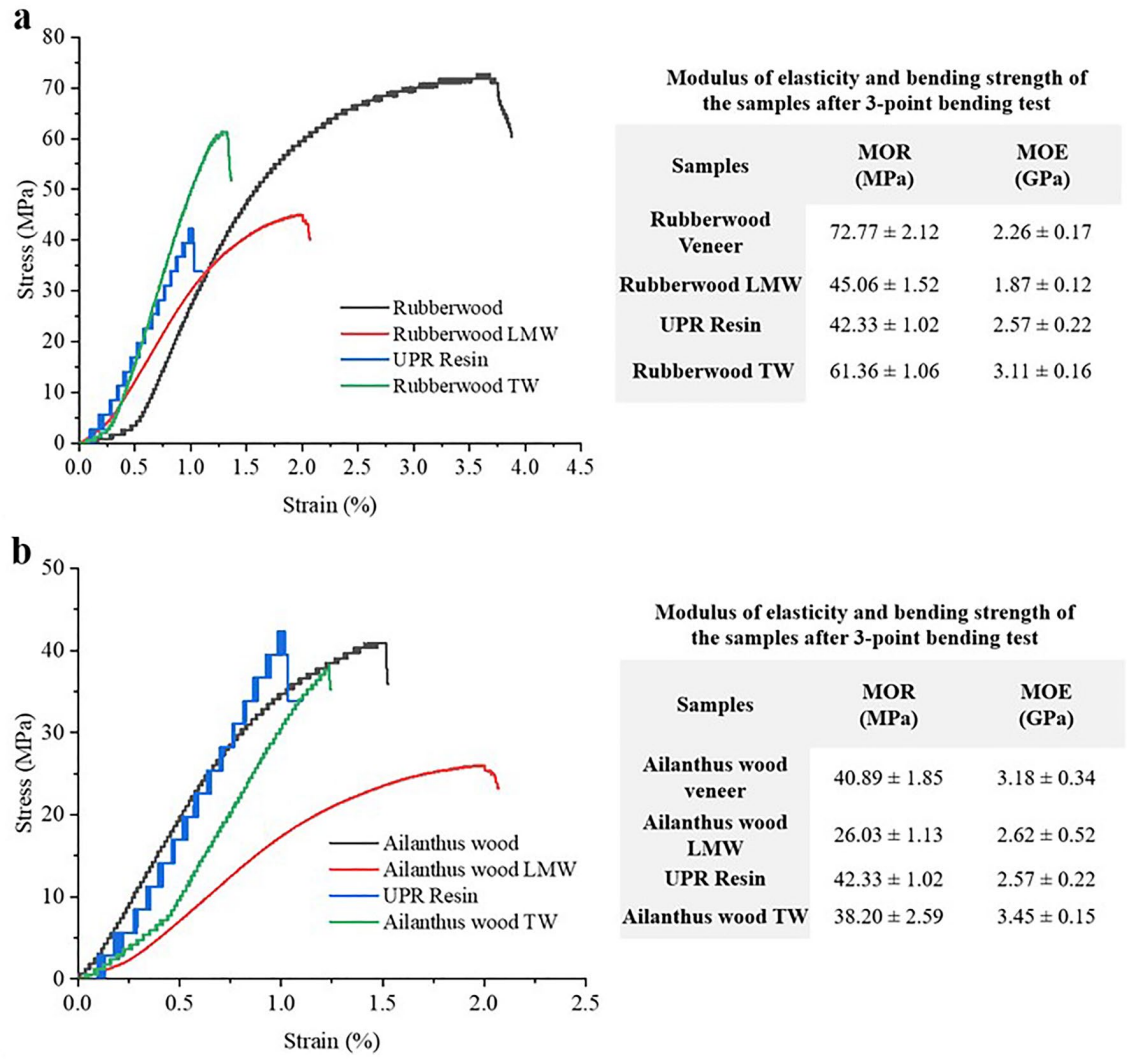
The comparison of mechanical property (MOE and MOR) of (**a**) rubberwood veneer, rubberwood LMW, UPR resin, and rubberwood TW; (**b**) ailanthus wood, ailanthus wood LMW, UPR resin, and ailanthus TW.

The modulus of rupture (MOR) values obtained after three-point bending test for rubberwood TW and Ailanthus TW were 61 MPa and 38 MPa (Fig. 5). Here, the combined impact of wood and polymer was visible in the mechanical properties of TW. The reduced mechanical strength of wood veneer after lignin removal process was compensated by the polymer reinforcement to provide the resultant composite a mechanical strength comparable to that of NW. In rubberwood TW, MOR approaches more towards the MOR values of NW with higher MOE values than that of the NW veneer. As ailanthus wood and UPR had MOR values of 41 MPa and 42 MPa, respectively, the TW obtained from the combination of both displayed an MOR of 38 MPa with a 3.45 GPa MOE. The variations in the flexural strength values of both TW types show that the mechanical characteristics of the wood species and their compatibility with the infused polymer both had profound influence on the strength characteristics of the final TW composite.

It is also worth noting that when compared to normal wood after the lignin modification process, the MOR values of delignified wood substrates have decreased to 38 percent in the case of rubberwood and 36 percent in the case of ailanthus wood. This reduction in mechanical properties of wood templates as a result of delignification was subsequently compensated through the infilled polymer reinforcement. The synergy and strengthening impact between delignified wood template and UPR resin in TW matrix is evident from its mechanical performance. Our findings show that, the mechanical properties of TW are highly dependent on the density of the species being used. The higher volume fraction of cellulose in rubberwood accounts for its enhanced MOR and MOE over low density ailanthus wood. Therefore, the TW composites made using rubberwood showed superior strength properties compared to that of ailanthus wood. In addition, the removal of constituent lignin, hemicellulose, and amorphous cellulose as a result of delignification enhances the porosity of wood substrate. This further reduces the density and mechanical integrity of the samples. Similarly in the case of both TW types, the proportionate change in density dependent cellulose volume fraction due to delignification has subsequently impacted their respective MOR and MOE.

The previous studies have reported a wide range of values regarding TW mechanical properties. So far results revealed that the mechanical strength of TW varies with respect to the extent of delignification, the strength of polymer, thickness, and orientation of the wood substrate, type of wood species etc.^10,14,18,27^. Zhu et al. obtained a fracture strength of 45.38 MPa along with a modulus of 2.37 GPa for transparent wood samples made using longitudinal wood sections of low-density basswood^14^. Meanwhile, we obtained a slightly lesser MOR and MOE values for TW fabricated using ailanthus wood of low density. The higher strength property displayed by rubberwood TW indicates the influence of wood species dependent physical strength on final TW. Similarly, strength values of longitudinal TW samples are known to vary from 31 MPa for balsawood TW^51^, to 174 MPa for birchwood TW^36^. It is obvious from the data that in all cases, the consequent strength reduction in wood substrates due to delignification were subsequently compensated by the strength of the polymer infused.

### Light diffusing ability of TW

**Figure 6:**
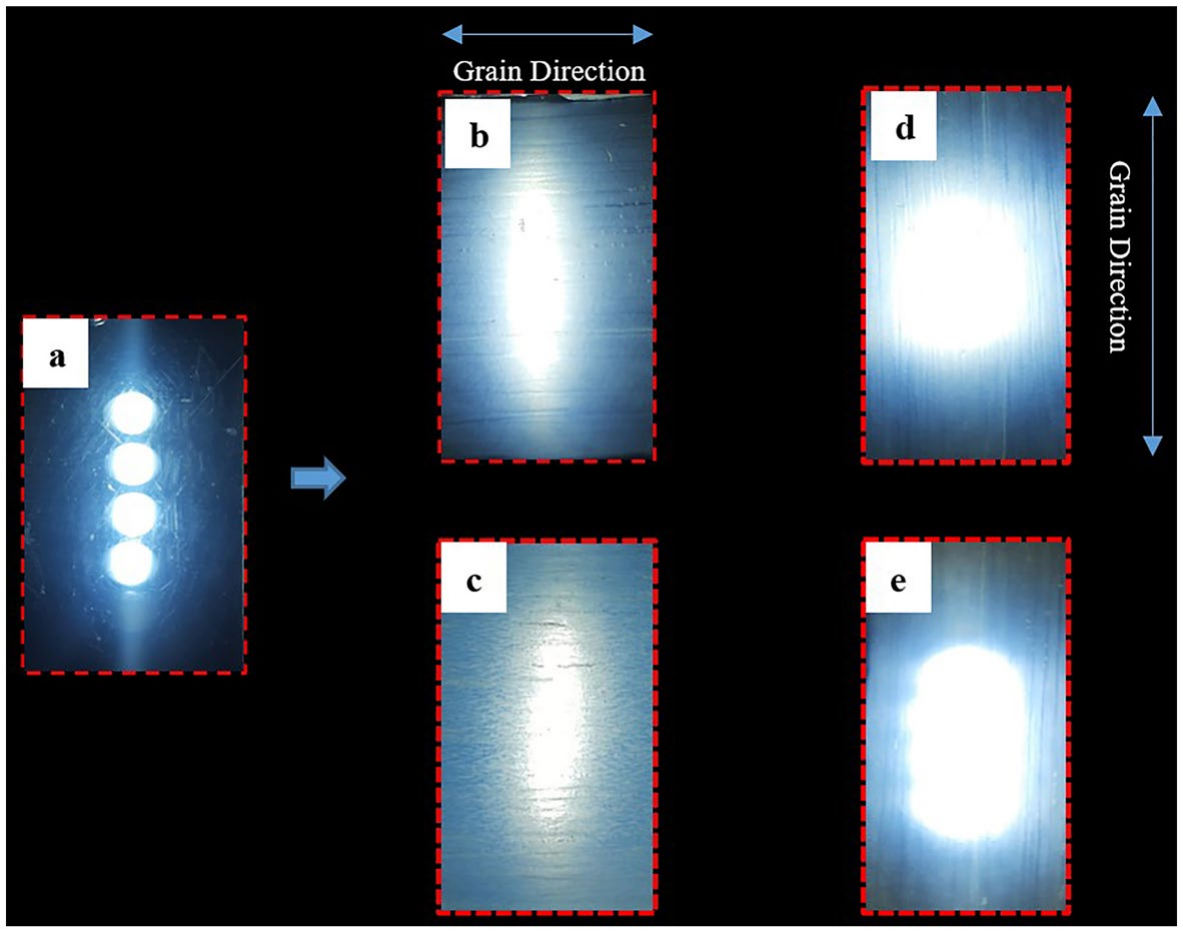
Demonstration of light diffusing property of TW: (**a**) white LEDs arranged 5 mm apart when viewed through pure UPR polymer sheet; light output through TW (**b**) rubberwood TW & (**c**) ailanthus TW with fibre direction perpendicular to the alignment of light source; light output through TW (**d**) rubberwood TW & (**e**) ailanthus TW with fibre direction parallel to the alignment of light source.

The application potential of TW has been demonstrated in a variety of circumstantial uses such as architectural lighting systems, interior decorations, solar collectors, organic display devices sensors, flame-retardant devices, and light screening systems, among others^52–55^. The light diffusing ability of TW prepared using two different timber species are demonstrated here. The set up involves a few numbers of LEDs connected in a series. Figure 6 shows the images when was lighted from behind the normal transparent UPR sheet (image a), and TW samples made using rubberwood and ailanthus wood (images b–e). The light diffusive ability of TW can be clearly seen. Besides, the degree of diffusion also varies with the orientation of TW which is exposed towards the LED light source. Similar fibre direction dependent light shaping behaviour has been previously reported for the flexible TW prepared using a combination of poplar wood and polyvinyl alcohol (PVA)^27^. A study on the anisotropic scattering ability of TW revealed that in TW most effective light scattering occurs when the light is polarised parallel to the cellulose fibres^12^. Yet another study discovered that, among other parameters, the mismatch in refractive indices (RI) between the polymer and the wood substrate had substantial impact on light scattering in TW^56^. Apart from that, the TW composites are suited for a variety of applications owing to the polymer induced hydrophobicity and shatterproof nature. These qualities make TW composites intriguing for a variety of eco-friendly opto-electronic applications, including energy-efficient glazing materials, smart windows, and others which also benefits the environment and fosters the growth of sustainable enterprises.

## Conclusions

Multipurpose TW composites were fabricated using wood veneers of two commercially important tropical timber species (rubberwood and ailanthus wood) through the two step processes of lignin modification and polymer impregnation. SEM micrographs indicated the homogeneous infiltration and polymerization of UPR polymer in cellulose rich substrates prepared from rubberwood and ailanthus wood veneers. Comparable optical transmittance of 59% for rubberwood TW and 55% for Ailanthus TW along with high haze of 94% and 89% were achieved from 2 mm thick veneers. Polymer infused TW samples showed strength properties at par with that of normal wood of same thickness. TW from low density ailanthus wood showed lesser mechanical strength compared to the TW obtained from medium density rubberwood. The application of wood based transparent input material as potential light diffusors has been demonstrated. In addition, the distinct light shaping behaviour of both TW consistent with the change in fibre direction was also illustrated. Furthermore, the study proposes the expanded utility of TW as light shaping devices, aesthetic lighting, optoelectronics, and chip on board overlays to deliver uniform lighting.

## Data Availability

The data that support the findings of this study are available from the corresponding author upon request.
